# Comprehensive review of materials, applications, and future innovations in biodegradable esophageal stents

**DOI:** 10.3389/fbioe.2023.1327517

**Published:** 2023-12-06

**Authors:** Yaochen Yang, Yuanyuan Yang, Zhipeng Hou, Tingting Wang, Peng Wu, Lufan Shen, Peng Li, Kai Zhang, Liqun Yang, Siyu Sun

**Affiliations:** ^1^ Department of Gastroenterology, Endoscopic Center, Engineering Research Center of Ministry of Education for Minimally Invasive Gastrointestinal Endoscopic Techniques, Shengjing Hospital of China Medical University, Shenyang, China; ^2^ Research Center for Biomedical Materials, Engineering Research Center of Ministry of Education for Minimally Invasive Gastrointestinal Endoscopic Techniques, Shengjing Hospital of China Medical University, Shenyang, China; ^3^ Liaoning Research Institute for Eugenic Birth and Fertility, China Medical University, Shenyang, China

**Keywords:** biodegradable esophageal stents, esophageal stricture, esophageal cancer, polydioxanone, poly (L-lactic-acid), magnesium alloy, drug-eluting stents

## Abstract

Esophageal stricture (ES) results from benign and malignant conditions, such as uncontrolled gastroesophageal reflux disease (GERD) and esophageal neoplasms. Upper gastrointestinal endoscopy is the preferred diagnostic approach for ES and its underlying causes. Stent insertion using an endoscope is a prevalent method for alleviating or treating ES. Nevertheless, the widely used self-expandable metal stents (SEMS) and self-expandable plastic stents (SEPS) can result in complications such as migration and restenosis. Furthermore, they necessitate secondary extraction in cases of benign esophageal stricture (BES), rendering them unsatisfactory for clinical requirements. Over the past 3 decades, significant attention has been devoted to biodegradable materials, including synthetic polyester polymers and magnesium-based alloys, owing to their exceptional biocompatibility and biodegradability while addressing the challenges associated with recurring procedures after BES resolves. Novel esophageal stents have been developed and are undergoing experimental and clinical trials. Drug-eluting stents (DES) with drug-loading and drug-releasing capabilities are currently a research focal point, offering more efficient and precise ES treatments. Functional innovations have been investigated to optimize stent performance, including unidirectional drug-release and anti-migration features. Emerging manufacturing technologies such as three-dimensional (3D) printing and new biodegradable materials such as hydrogels have also contributed to the innovation of esophageal stents. The ultimate objective of the research and development of these materials is their clinical application in the treatment of ES and other benign conditions and the palliative treatment of malignant esophageal stricture (MES). This review aimed to offer a comprehensive overview of current biodegradable esophageal stent materials and their applications, highlight current research limitations and innovations, and offer insights into future development priorities and directions.

## Graphical Abstract

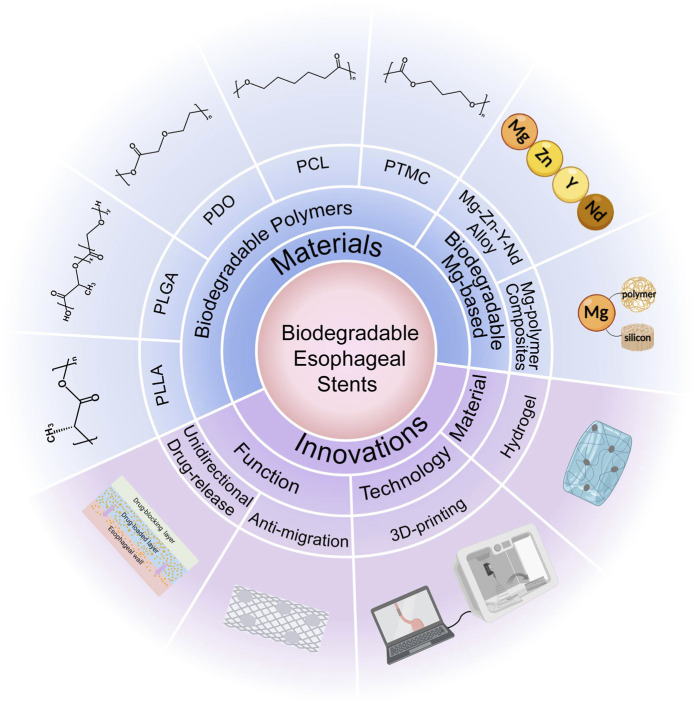

## 1 Introduction

Esophageal stricture (ES) results from prevalent clinical conditions and is categorized into two primary types: benign esophageal stricture (BES) and malignant esophageal stricture (MES). The most frequently encountered BES subtype is peptic strictures (Desai et al., 2020), typically stemming from uncontrolled gastroesophageal reflux disease (GERD), radiation-induced strictures, and iatrogenic injuries (De Wijkerslooth et al., 2011; Liu et al., 2022). In contrast, MES is predominantly associated with esophageal neoplasms and extraneous malignancies that compress the esophagus (Siersema, 2008). Upper gastrointestinal endoscopy serves as the preferred diagnostic modality for identifying ES and its root causes (Siersema, 2008). Patients commonly present with dysphagia once the stenosis reduces the esophageal lumen by 50% (Adler and Siddiqui, 2017). ES can trigger various adverse consequences such as malnutrition, weight loss, acid-base imbalance, and hypoalbuminemia. Consequently, timely intervention is imperative, particularly when these adverse conditions manifest.

Current treatments for ES encompass various modalities, including drug therapy, surgical interventions, endoscopic esophageal dilation, and endoscopic stent placement, among others (Sehgal and Sami, 2021). Pharmacological treatment entails the administration of proton pump inhibitors (PPI) for managing GERD (Peng et al., 2023). Additionally, endoscopy-guided infusion of adrenocorticotropic and cytotoxic drugs into the stenosis site serves to inhibit fibrosis and scar formation in damaged tissue, thereby alleviating scarring-induced permanent ES (Zein et al., 1995). However, these drugs may prove ineffective, lead to disease recurrence upon discontinuation, and even give rise to significant side effects, limiting their standalone usage in current clinical practice. Esophagectomy becomes necessary in severe ES unresponsive to conservative measures such as endoscopic treatment.

In contrast to surgery, endoscopy boasts advantages such as reduced trauma, a high cure rate, and rapid recovery (Chen et al., 2022). Esophageal dilation is one of the most practical and effective techniques for managing BES, with a success rate exceeding 80%–90% (Siersema, 2019). Balloon dilation is the preferred method for achieving this outcome. Moreover, endoscopic dilation of the esophagus, whether by employing a Savary (bougie) or a balloon dilator, serves as the initial step in managing refractory benign esophageal stricture (RBES) (Sehgal and Sami, 2021). Balloon dilation in ES yields immediate relief; however, it cannot ensure sustained dilation. Patients with RBES often require repeated dilations, which can be inconvenient and necessitate high compliance rates (Siersema, 2019). Additionally, balloon dilation can serve as a complementary procedure to esophageal stent insertion by performing several balloon dilations before stent placement, which prepares the stricture for the smooth passage of the stent delivery device. Esophageal stent insertion using endoscopy involves the deployment of a stent to support the stricture and maintain radial pressure, facilitating the remodeling of the stenosis and its surrounding tissue.

Indications for esophageal stent placement include BES, primarily resulting from GERD-induced chronic inflammation (Desai et al., 2020), scarred strictures following chemical burns, and stenosis after post-surgical anastomosis or endoscopic resection procedures such as endoscopic submucosal dissection (ESD) and endoscopic mucosal resection (EMR) (Siersema, 2019). Stent placement is also indicated for MES, including unresectable esophageal or gastric cardiac cancer, local recurrence at the anastomosis site following esophagectomy, and strictures arising after radiation therapy for esophageal cancer. Additionally, stents may be employed to address other conditions such as esophagotracheal fistulas and esophageal ruptures, among others. Among these indications, MES is frequently linked to esophageal cancer, which encompasses two distinct pathological and epidemiological subtypes: esophageal squamous cell carcinoma (ESCC) and esophageal adenocarcinoma (EAC) (Smyth et al., 2017). Esophageal cancer is a global health concern, ranking as the sixth most prevalent cause of cancer-related fatalities each year. Over 50% of patients with esophageal cancer experience dysphagia due to malignant obstruction. Treatment for esophageal cancer hinges on patient-specific factors and tumor characteristics, particularly the TNM stage, which is usually identified using imaging methods and histopathologic biopsies (Liu et al., 2023; Matsunami et al., 2023). Early-stage tumors are typically amenable to endoscopic resection, whereas locally advanced cancers necessitate surgical resection, neoadjuvant chemoradiotherapy, chemotherapy, radiotherapy, or a combination thereof (Chen et al., 2023). However, a considerable proportion of patients present with advanced, unresectable tumors at the time of diagnosis. Treatment objectives in such cases primarily constitute restoring oral food intake and enhancing quality of life (Rozanes et al., 2002). Notably, over half of the patients pursuing curative treatment will experience tumor recurrence, necessitating palliative care for the majority of individuals in the long term (Smyth et al., 2017). Consequently, improved treatment options for esophageal cancer should be explored.

Esophageal stenting has a rich history that dates back over a century. Its origins can be traced back to Symonds’ pioneering proposal in 1885 regarding the advantages of esophageal stent placement. In 1977, Arkinson introduced a cylindrical plastic tube to address inoperable esophageal tumors, marking the inception of the clinical use of esophageal stents. Frimberger first treated ES with a spiral metal stent in 1983, and Domschke applied the Wallstent to treat cancer-related ES in 1990. A pivotal moment occurred in 1996 when Goldin et al. (1996) introduced an esophageal stent constructed from poly-L-lactide (PLLA), which was both biodegradable and self-expandable (Sehgal and Sami, 2021). Subsequently, a diverse array of esophageal stents employing various materials and functions emerged thereafter. Currently, several types of esophageal stents exist (Wang et al, 2021b): self-expandable metal stents (SEMS), self-expandable plastic stents (SEPS), biodegradable stents (BDS), and hybrids that combine elements of the aforementioned stents, such as SEMS with an anti-reflux valve, DES, and radioactive SEMS, among others. Among these options, SEMS has emerged as the preferred and efficacious choice for managing BES and MES (Yang et al., 2016). The most commonly employed endoscopic therapeutic approach for palliation involves the placement of esophageal stents (Rabenstein, 2015), particularly in cases involving patients with cancer and limited life expectancy who require rapid relief from dysphagia or those unresponsive to chemoradiotherapy. However, SEMS has certain limitations. The necessity of stent removal after stenosis resolution in certain benign conditions and the potential complications of metal materials such as bleeding, retrosternal pain, esophageal fistula, tissue ingrowth, and restenosis (Song et al., 1997; Ackroyd et al., 2001; Evrard et al., 2004; Yang et al., 2016), has rendered SEMS less than ideal for ES treatment, particularly BES treatment. SEPS offers advantages, such as reduced tissue trauma, minimal tissue proliferation, and ease of removal (Verschuur et al., 2008; Rabenstein, 2015); however, its long-term effectiveness is disappointing. SEPS often requires reintervention and carries a higher complication rate than SEMS, including complications such as stent migration (Conio et al., 2007; Ham and Kim, 2014; Fuccio et al., 2015). Moreover, its infrequent application for the management of malignant dysphagia can be linked to factors such as the wide diameter of the stent deployment catheter, the complex and unwieldy assembly and operation, and its heightened mobility (Adler and Siddiqui, 2017).

Over the past 3 decades, biodegradable materials, including synthetic polyester polymers and magnesium (Mg)-based alloys (Wu et al., 2012; Yuan et al., 2016; Liu et al., 2022), have garnered substantial attention owing to their distinct advantages when compared to SEMS and SEPS. An ideal biodegradable esophageal stent should possess exceptional mechanical properties, compliance, and histocompatibility, ultimately undergoing complete degradation after providing a period of mechanical support. The resulting degradation products should have no adverse effects on human health (Yang et al., 2016). Considering that a stent’s degradability is pivotal in mitigating the risk of severe long-term complications, stents crafted from biodegradable materials are highly favorable for managing benign conditions (Tamai et al., 2000; Saito et al., 2004; Tanaka et al., 2007). The critical factor in developing an outstanding esophageal stent lies in the selection of appropriate biodegradable materials. Consequently, our discussion centered on the materials and applications of biodegradable esophageal stents, highlighted the current research challenges, and offered insights into future development priorities and directions.

## 2 Materials used in biodegradable esophageal stents

A diverse array of effective stents constructed from biodegradable materials, encompassing synthetic polyester polymers and Mg-based alloys, has found widespread application in the cardiovascular, airway, esophageal, and urinary systems in contemporary medical practice (Li et al., 2023). In 2006, the US Food and Drug Administration (FDA) approved the use of several synthetic polymers in medical devices (Kohn et al., 2007), including poly-L-lactide (PLLA), poly (lactic-co-glycolic acid) (PLGA), polydioxanone (PDO), poly (ε-caprolactone) (PCL) and poly (1, 3-trimethylene carbonate) (PTMC). Presently, these materials, their copolymers, and their composites constitute the forefront of ongoing research efforts in this field. Notably, the ultimate products of their degradation within the human body are water and carbon dioxide, posing no harm to human health (Li et al., 2023).

Two notable options among the biodegradable polymer stents currently accessible in clinical practice are the PLLA-BD stent (Marui Textile Machinery Co., Ltd., Osaka, Japan), comprising braided PLLA monofilaments (Tanaka et al., 2007), and the SX ELLa-BD stent (Ella-CS, Hradec Kralove, Czech Republic), composed of PDO, which is a type of surgical suture material (Stivaros et al., 2010) (Table 1).

**TABLE 1:** Comparison of biodegradable polymer stents currently available for clinical use.

	PLLA-BD stent (Tanaka-Marui stent; Marui Textile Machinery Co., Ltd., Osaka, Japan)	SX ELLa-BD stent (Ella-CS, Hradec Kralove, Czech Republic)
Materials	Knitted PLLA monofilament	Woven PDO monofilament
Initial application time	2006	2008
Length and diameter	Designed according to the esophageal lesion of each individual patient	Size: 18, 20, 23, 25 mm Length: 60, 80, 100, 135 mm
Procedure for stent insertion	One end of the PLLA stent is to be tied into a 5 mm diameter by silk sutures and then fitted over an endoscope	Manually load the stent into the delivery system and position it on the basis of the metal stent markers at both ends. The stent can be fully expanded to its designed diameter after 24–48 h in the body
Biodegradable time	3–6 months (acidic environment will accelerate its degradation)	2–3 months (acidic environment will accelerate its degradation)
Advantages	Excellent expansion capacity, high radial force (117 gf, which is comparable to commercially available metallic stents), low incidence of stent-related complications	Superior flexibility and biocompatibility
Disadvantages	Low flexibility, early spontaneous migration	Lower mechanical stability compared to PLLA stent, prone to complications

Metals containing elements naturally present in the human body are deemed biocompatible, rendering them suitable for the fabrication of biodegradable stents. Notably, Mg, iron (Fe), zinc (Zn) and their respective alloys are among this category. These metals and alloys offer sustained radial support over a defined period of time, while undergoing spontaneous degradation within the body (Bowen et al., 2016; Liu et al., 2022). Therefore, they can help minimize inflammation from local tissue hyperplasia or prolonged physical stimulation (Ragunath, 2008), precluding the need for secondary stent removal (Chen et al, 2019a). Among these biodegradable alloys, Mg holds particular significance as an essential trace element and a structural component of tissues (Moravej and Mantovani, 2011). Furthermore, Mg is recognized as a non-carcinogenic element (Xu et al., 2007). Additionally, Mg-based alloys exhibit superior strength, specific stiffness, corrosion resistance, and processability (Bowen et al., 2016; Liu et al., 2022). The following section presents a comprehensive review of synthetic polyester polymers, Mg-based alloys and their related substances, focusing on materials, applications and innovative developments.

### 2.1 Biodegradable polymers

#### 2.1.1 PLLA

Polylactic acid (PLA) is an aliphatic polyester that is biodegradable, bio-based, and can be entirely sourced from renewable materials such as corn, potatoes, sugar cane, and similar resources (Yusoff et al., 2021). PLA demonstrates commendable biocompatibility, showcasing notable attributes such as strength, toughness, and plasticity. PLA hydrolysis yields products such as lactic acid, which is absorbed and metabolized by the human body, ultimately generating carbon dioxide and water that can be efficiently expelled from the body without leaving any residues. Lactic acid is a chiral molecule that exists in L and D isomers; therefore, four types of PLA arise, including isotactic PLA (PLLA), isotactic poly-D-lactic acid (PDLA), and atactic and syndiotactic poly-D, L-lactic acid (PDLLA) (Lasprilla et al., 2012; Zhou et al., 2021). Among them, PLLA, which is one of the stereo configurations of PLA (Wu et al., 2023), is a biodegradable material produced through the polymerization of L-lactic acid. Its versatility has led to its application in surgical sutures, bone screws, and tissue engineering scaffolds (Saito et al., 2007; Tanaka et al., 2007; Shuai et al., 2019; Liu et al., 2020; Shuai et al., 2021; Yin et al., 2022), and an array of stents designed for use in coronary arteries, urinary systems, bronchial passages, and biliary tracts (Tamai et al., 2000; Saito et al., 2004; Isotalo et al., 2006; Song et al., 2022).

In 1996, Goldin et al. (1996) documented the groundbreaking experience with a novel stent that possessed the unique features of self-expansion and biodegradability. The stent was constructed as a coil spring using a solitary strand of PLLA (InStent Inc) and was designed to treat BES. Furthermore, Fry and Fleischer (Fry and Fleischer, 1997) pioneered the application of PLLA-BDS (EsophaCoil; InStent, Eden Prairie, MN, United States) in the United States, particularly for BES caused by radiation-induced injury. This marked a significant expansion of the knowledge and experience in the application of biodegradable esophageal stents.

A limited amount of research has been conducted on the clinical application of PLLA stents (Table 2). In 2006, Tanaka et al. (2007) introduced a new biodegradable Ultraflex-type stent, utilizing machine-knitted polylactic acid monofilaments (Marui Textile Machinery Co., Ltd., Osaka, Japan), to treat two patients with benign strictures in the upper gastrointestinal tract. These stents were layered with a water-soluble contrast agent (gastrografin) to enhance visibility during placement and were subsequently guided into position using a combination of fluoroscopy and endoscopy. A thread was used to fasten the stent’s tip and diminish its diameter, resulting in easier navigation through narrower sections. In case 1, a 19-year-old woman had engaged in a suicide attempt by consuming caustic potash, resulting in the development of ES 2 weeks later. In case 2, a 75-year-old man developed stenosis at the anastomotic site after undergoing surgical resection for esophageal cancer. Balloon dilatation therapy was attempted multiple times; however, neither patient experienced relief from their dysphagia. Consequently, both individuals received the same treatment approach. After two sessions of balloon dilatation, the stents were meticulously placed under combined fluoroscopic and endoscopic guidance, resulting in immediate improvement of their strictures. Remarkably, the stents naturally migrated and were excreted with feces via bowel movements at 10 and 14 days post implantation for cases 1 and 2, respectively, with no associated complications. In case 1, there was no recurrence at the 6-month follow-up examination. However, mild restenosis occurred 1 week later in case 2 but did not necessitate further dilatation therapy during the subsequent 6 months of follow-up.

**TABLE 2:** Clinical studies of PLLA-BDS.

Author	Year	Study design	n	Indication	Follow-up	Migration (%)	Complication	Clinical success (%)	Reference
Tanaka et al.	2006	Clinical trial	2	BES (corrosive stenosis and postoperative anastomotic stenosis)	6 months	10 days and 2 weeks, respectively	0	2 (100%)	Tanaka et al. (2007)
Saito et al.	2007	Case report	13	BES (corrosive stenosis, stricture after surgical resection of esophageal cancer, preventive placement for post-ESD stenosis)	7 months to 2 years	10 (77%): spontaneous migration within 10–21 days	0	13 (100%)	Saito et al. (2007)
Saito et al.	2008	Case report	2	BES following ESD	6 months	0	0	2 (100%)	Saito et al. (2008)
Mochizuki et al.	2012	Case report	1	Recurrent esophageal cancer after chemoradiotherapy	1 month	0	obstruction	1 (100%)	Mochizuki et al. (2012)

Saito et al. (2007) used PLLA-BDS (Marui Textile Machinery Co., Ltd., Osaka, Japan) to treat BES in 2007. These stents were composed of PLLA monofilaments (molecular mass 183 kD, diameter 0.23 mm), with a machine-knitted design akin to ultra-flex metallic stents. These stents were applied in various cases, including BES resulting from anastomosis following surgical resection for esophageal cancer, corrosive liquid ingestion in suicide attempts, and the preventive insertion of biodegradable stents for post-ESD strictures. Each PLLA-BDS was custom-tailored to the specific characteristics of the patient’s esophageal condition and meticulously fitted using an endoscope. Remarkably, spontaneous early migration was observed in 77% (10/13) of these stents, possibly attributed to stent degradation, leading to loss of patency. No signs of restenosis were observed during the follow-up period, which ranged from 7 months to 2 years. This study demonstrated the utility of biodegradable stents in managing BES, with a specific focus on their role in preventing post-ESD stenosis. Presently, a rising number of patients diagnosed with early-stage esophageal cancer are opting for full resection through minimally invasive endoscopic techniques such as ESD. However, strictures tend to develop when the mucosal defect encompasses >75% of the esophageal circumference (Oyama et al., 2005), and endoscopic dilation or stenting may be required in these cases. Saito et al. (2008) also reported two cases of using PLLA stents to successfully improve BES following ESD in patients with early ESCC and repeated failed balloon dilatation.

Additionally, researchers reported a case of a patient with recurrent ESCC following chemoradiotherapy (CRT) (Mochizuki et al., 2012). Biodegradable esophageal stents composed of PLLA monofilaments (Saito et al., 2008) were promptly inserted on the same day of ESD to prevent stenosis resulting from the mucosal defect. This study demonstrated the potential efficacy of combining ESD with biodegradable stents, introducing a viable approach for managing recurrent ESCC following CRT and offering a promising avenue for enhancing the treatment outcomes and prognosis of esophageal malignancies.

Tanaka et al. (2007) substantiated the superior radial force of their biodegradable stent, which was constructed from polylactic acid monofilaments, with higher radial force measurements than commercially available metal stents. Moreover, this stent exhibited a marked reduction in various complications typically associated with the use of metal stents in BES treatment. Nonetheless, these studies also highlighted a potential drawback of PLLA stents, namely, the occurrence of early migration. PLLA-BDS take months to degrade; therefore, the migration risk arises from the gradual loss of radial force before complete degradation. No complications were observed with the softened stents during defecation; nevertheless, the ideal approach is to prevent these early displacements to ensure the stents effectively treat strictures. The research group speculated on the necessity of securing the stents in place for a minimum of 2 weeks, which represents the required time for healing inflammatory changes in the lesions. No bowel obstruction caused by PLLA stents was reported in the aforementioned cases; however, vigilant attention should be directed towards the potential risk of bowel obstruction from migrated stents.

In summary, PLLA stents exhibit noteworthy attributes, including excellent expansion capacity, high radial force, and the ability to biodegrade within 3–6 months when exposed to an acidic environment (Yang et al., 2016). These qualities contribute to a low incidence of stent-related complications. However, their inherent brittleness limits their flexibility (Han et al., 2018). Furthermore, PLLA stents tend to experience a relatively high rate of early spontaneous migration as they gradually degrade, necessitating vigilant monitoring for potential complications such as intestinal obstruction.

#### 2.1.2 PLGA

PLGA is a biodegradable and biocompatible polyester copolymer constructed by the polymerization of lactic acid and glycolide (Stevanović et al., 2009; Ma et al., 2011) and has emerged as a principal biodegradable polymer over the past few decades. It serves as the primary polymer for drug delivery (Shakya et al., 2023) and plays a pivotal role in diverse clinical applications (Liu et al., 2016). The hydrolytic breakdown of PLGA yields lactic acid and glycolic acid (Ma et al., 2011; Song et al., 2022), which are ultimately converted into carbon dioxide and water and subsequently eliminated through the body’s normal metabolic processes. PLGA is renowned for its low toxicity and rapid degradation and is widely used in the treatment of bone defects, tissue engineering, surgical sutures, and drug release systems (Stevanović et al., 2009; Fredenberg et al., 2011; Lanao et al., 2011; Babilotte et al., 2021; Jin et al., 2021).

Liu et al. (2016) designed a novel biodegradable stent comprising three curved segments of covered metal mesh linked together by biodegradable PLGA threads. As these PLGA threads degraded, the stent disintegrated and safely passed into the stomach. This study investigated the novel stent’s attributes using *in vitro* assessments, including evaluations of radial forces, pH levels, and morphology, among others. Additionally, they conducted *in vivo* animal studies, involving the insertion of these stents into the narrow middle esophagi of rabbits, which had incurred damage from alkali burns. The braided design of these stents enabled a reduction of the radial force, reaching 0 N (Hirdes et al., 2013), without any associated stent-related complications. This innovative design, namely, combining the strengths of metallic stents in terms of radial force with the biodegradability of biodegradable stents, holds considerable promise and offers a novel avenue for the future development of biodegradable stent technology.

Prior studies have demonstrated the promise of utilizing PLGA as a surface coating, particularly on Mg or Zn alloys, owing to its commendable attributes including robust biocompatibility, pronounced hydrophobicity, and proficient drug-loading capabilities (Wang et al., 2006; Zhu and Braatz, 2015; Dou et al., 2023). The versatile applications of PLGA coatings have been extensively explored by researchers in various stent domains, including cardiovascular stents (Fang et al., 2021; Dou et al., 2023), ureteral stents (Antonowicz et al., 2021), and gastrointestinal stents (Kwak et al., 2017), among others.

**FIGURE 1:**
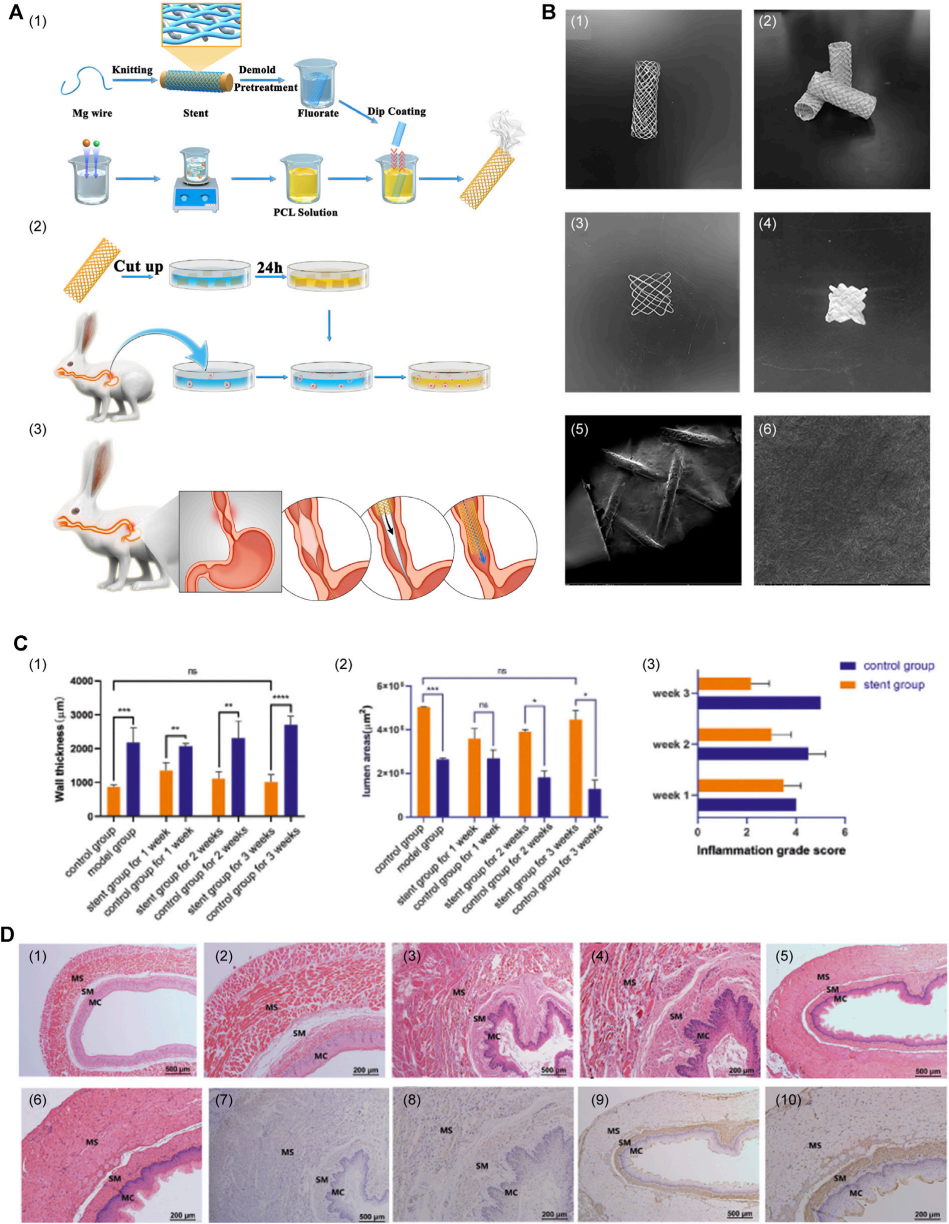
**(A)** Primary procedure and methods. (1) Construction of PLGA-coated drug-eluting Mg stents. (2) Experiments on *in vitro* degradation, drug release, and cytotoxicity. (3) Insertion of the stent. **(B)** Macrostructure and surface characteristics of the stent. (1) The Mg alloy bare stent. (2) The PTX-PLGA-coated Mg-based stent. (3) The 1 cm × 1 cm stent sheet of bare stent. (4) The 1 cm × 1 cm stent sheet of the coated stent. (5 and 6) SEM images of the surface characteristics. **(C)** (1–3) Wall thickness of the esophagus, lumen area, and degree of inflammation between the stent group and sham stent group. **(D)** (1 and 2) Esophageal tissue of normal control rabbits. (3 and 4) Imaging of esophageal tissue over a period of 3 weeks after successful modeling, with no stent insertion. (5 and 6) Imaging of esophageal tissue 3 weeks after stent insertion following the successful modeling. (7 and 8) Immunohistochemical staining images of stent group and (9 and 10) the sham stent group. Reproduced with permission from ref [(Liu et al., 2022)]. Copyright 2022 Acta Materialia Inc.

Liu et al. (2022) designed and tested a groundbreaking Mg-based braided stent coated with PLGA infused with paclitaxel (PTX) to avoid secondary removal by endoscopic procedures and inhibit restenosis of the esophagus (Figure 1). This ingeniously engineered stent was designed to undergo self-degradation within the body after ensuring esophageal patency for a specified duration, precluding the necessity for subsequent removal procedures. In addition, the stent’s surface was coated with anti-proliferative drugs, which can further inhibit local tissue growth and extend the unobstructed period of esophageal function. The safety and effectiveness of this combined stent have been validated through *in vitro* and *in vivo* testing, whereas its sustained applicability and worthiness await further evaluation in subsequent studies. In conclusion, this stent effectively amalgamated the merits of biodegradable alloys and polymers, thereby compensating for their limitations. This innovation presents a promising and novel avenue for future research and development in biodegradable stent technology.

#### 2.1.3 PDO

PDO is a biodegradable polymer with a semicrystalline structure, belonging to the polyester family. Its degradation occurs through the random hydrolysis of ester bonds within its molecular structure, with glyoxylic acid, a degradation product, primarily serving as a precursor for oxalic acid and playing a role as an intermediate in the transformation of glycolic acid into glycine. All of PDO’s degradation products and intermediates are non-toxic (Repici et al., 2010). A significant milestone was achieved when PDO gained FDA approval in 1981 for its use in biodegradable sutures (Bartholomew, 1981). PDO can be knitted into tubes of the desired shapes and dimensions, and stents composed of PDO have been inserted into the bronchus, trachea, intestine, and bile duct, in addition to the esophagus (Lischke et al., 2011; Rejchrt et al., 2011; Siiki et al., 2017). PDO’s utility extends even further into domains such as tissue engineering (Boland et al., 2005; Okata et al., 2014; Goonoo et al., 2015), bone regeneration (Saska et al., 2021), and drug delivery systems (Goonoo et al., 2015; Bottino et al., 2019), among others. The SX ELLa-BD stent (Ella-CS, Hradec Kralove, Czech Republic) is a biodegradable esophageal stent constructed using commercially accessible PDO absorbable surgical sutures (Repici et al., 2010). This stent is Conformité Européenne approved and is indicated for use in benign strictures, including digestive, erosive, anastomotic, and post-radiation strictures (van Hooft et al., 2011). The ELLa-BDS comes in various sizes, with stent body diameters ranging from 18 to 25 mm and lengths from 60 to 135 mm when fully deployed. It should be compressed and installed onto a 9.4 mm (28F) delivery system prior to clinical use. Regarding performance, this stent exhibits a relatively high axial force and initially low radial force, which gradually decreases to 0 N (Hirdes et al., 2013). Its structural integrity and radial expansion strength remain stable for a period of 6–8 weeks, after which disintegration occurs within 11–12 weeks post implantation. Notably, low pH can accelerate its degradation; therefore, the use of a PPI is advised to extend the integrity of the stent (Yang et al., 2016).

**FIGURE 2:**
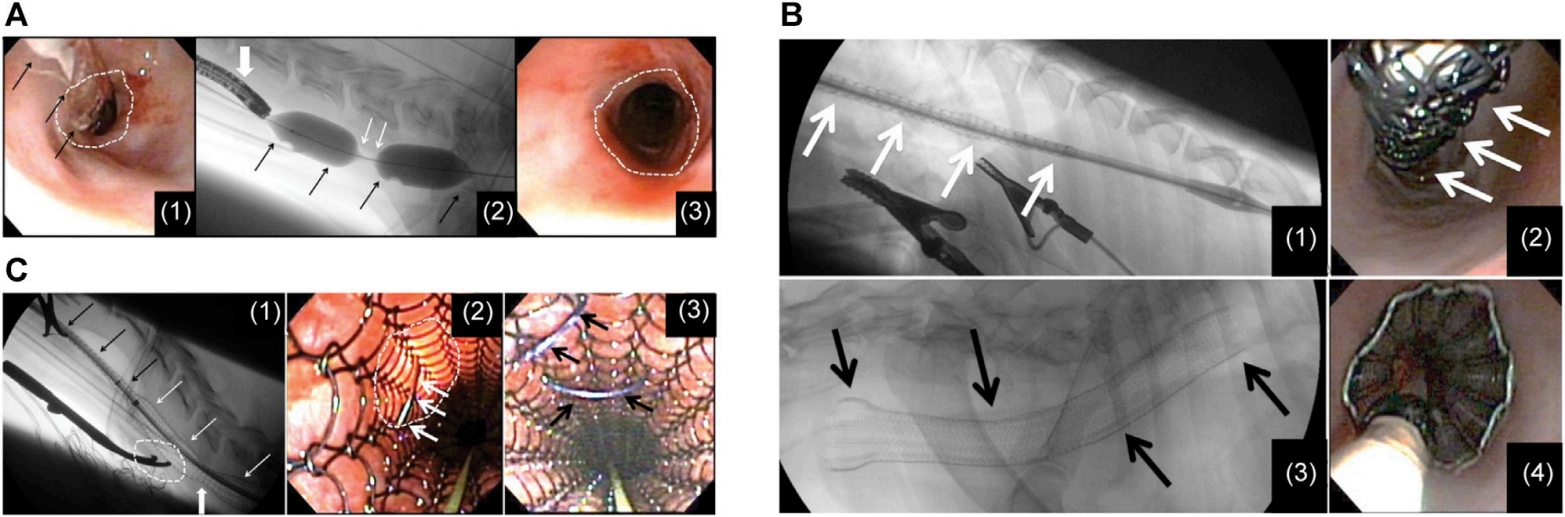
**(A)** (I) A flexible endoscope and dilatation balloon were inserted into the esophagus to visualize the stricture. Balloon dilatation was performed to allow the endoscope to pass through. (II) The stent in a restricted state was advanced with the guidance of fluoroscopy. (III) Following the deployment of the stent, the upper thoracic stricture was resolved at the site of stent constriction, revealing a clear and unobstructed esophageal passage. **(B)** (I) A restricted stent was advanced through an esophageal constriction within the thoracic area. (II) Endoscopic image of the stent while it was constrained before deployment. (III) Lateral fluoroscopic image following the stent insertion. (IV) The endoscopic picture taken right after stent insertion, illustrating effective esophageal wall apposition. **(C)** (I) Lateral fluoroscopic image showing an esophageal stent in a state of partial deployment and partial constriction. (II) Endoscopic view showing a luminated area where the surgical approach had been performed and the suture needle passing through the esophageal wall and engaging the stent. (III) Endoscopic view after 2 polypropylene sutures had been placed to tack the stent to the esophageal wall. Reproduced with permission from ref [(Lam et al., 2013)]. Copyright 2013 American College of Veterinary Internal Medicine.

Several preclinical studies on ELLa-BDS have been conducted. For example, Battersby and Doyle (2010) used a biodegradable PDO self-expanding stent to treat BES in a cat in 2010. A fluoroscopic examination revealed the complete disappearance of the stent 4 months after placement, with no signs of obstruction. In 2012, Pauli et al. (2012) reported ELLa-BDS’s potential to alleviate the development of severe strictures after performing circumferential endoscopic esophageal mucosectomy (EEM) in a porcine model. The findings indicated that the BDS group exhibited significantly extended survival compared to the control group (9.2 weeks vs. 2.4 weeks). However, the stent was unable to entirely prevent the development of high-grade strictures, and the timing of stenosis formation appeared to be linked to the gradual loss of radial force and stent disintegration. A retrospective review (Lam et al., 2013) of records for six dogs with RBES revealed initial improvements in dysphagia following ELLa-BDS insertion (Figure 2). Nonetheless, close monitoring is essential owing to the potential complications, including regurgitation, stent migration, and restenosis.

ELLa-BDS is recognized as the first commercially available biodegradable esophageal stent (Vandenplas et al., 2009; Repici et al., 2010; Stivaros et al., 2010) and has been extensively employed in clinical studies to evaluate its effectiveness and safety in the treatment of BES (Table 3). Previous studies have shown that ELLa-BDS offers a practical approach to managing RBES by reducing the need for repetitive endoscopic dilations and cutting treatment costs (Dhar et al., 2009; Repici et al., 2010; van Boeckel et al., 2011; Muñoz et al., 2013). A single BDS placement provides only temporary relief; therefore, the sequential placement of stents emerges as an excellent option to preclude the need for serial dilations (Hirdes et al, 2012a). Moreover, the commendable efficacy and safety of ELLa-BDS in addressing corrosive esophageal strictures and dysphagia from benign anastomotic esophageal strictures have also been demonstrated (Vandenplas et al., 2009; van Hooft et al., 2011). In a noteworthy case (Basha et al., 2013), researchers employed ELLa-BDS to treat a patient with corrosive (sulfuric acid-induced) esophageal and pyloric strictures. The patient underwent transhiatal esophagectomy with colonic transposition 6 months after stent insertion. The examination of the resected esophageal specimen marked the first-ever report of histological evidence in a human case, confirming ELLa-BDS’s excellent biocompatibility, minimal tissue response, and complete degradation capabilities. A cohort study conducted in 2016 (McCain et al., 2016) revealed that ELLa-BDS offered a probability of long-term symptom relief that exceeds 50% for patients with benign conditions. Regarding patients that required re-intervention, the duration of symptom-free periods and the time until re-intervention significantly surpassed expectations in comparison to SEMS, SEPS, or dilation procedures. In 2018, Walter et al. (2018) conducted a comparative study between early dilation and ELLa-BDS insertion in the treatment of recurrent BES and concluded that ELLa-BDS insertion reduced the need for repeated dilations and prolonged the time before recurrent dysphagia, in contrast to dilation. In 2022, a non-randomized, single-arm prospective trial (Yano et al., 2022) conducted across eight institutions provided compelling evidence that ELLa-BDS was indeed effective in treating RBES and demonstrated a satisfactory safety profile. Furthermore, a recent systematic review and proportion meta-analysis conducted by Kailla et al. (2023) in 2023 demonstrated that ELLa-BDS’s high technical sophistication and moderate clinical efficacy support its use in the management of adult BES. However, further evidence is required to substantiate its advantage over alternative approaches such as endoscopic dilation.

**TABLE 3:** Clinical studies of ELLa-BDS.

Author	Year	Study design	n	Indication	Follow-up	Migration (%)	Complication	Clinical success (%)	Reference
Orive-Calzada et al.	2009	Case report	1	BES (postoperative anastomotic stenosis)	3 months	0	Severe epithelial hyperplasia stenosis	0	Orive-Calzada et al. (2009)
Stivaros et al.	2010	Retrospective	2	1 BES (a peptic stricture in the distal esophagus) and 1 MES (a carcinoma at the gastro-esophageal junction)	3–4 months	2 (100%)	Transient nausea	2 (100%)	Stivaros et al. (2010)
Hair et al.	2010	Case report	1	BES (achalasia)	8 weeks	0	Severe hyperplasia stenosis, stent collapse, regurgitation and moderate weight loss	0	Hair and Devonshire (2010)
Jung et al.	2010	Case report	1	RBES (stenosis after CRT for a ESCC)	4 weeks	0	Tracheoesophageal fistula	0	Jung et al. (2010)
van Hooft et al.	2011	Prospective	10	BES (benign anastomotic esophagogastric strictures)	6 months	2 (20%)	Tissue hyperplasia, reobstruction	6 (60%)	van Hooft et al. (2011)
van Boeckel et al.	2011	Prospective	18	RBES (peptic, anastomotic, post radiation, caustic and other strictures)	166 days (range 21–559 days)	4 (22%)	Hemorrhage, severe retrosternal pain	6 (33%)	van Boeckel et al. (2011)
Hirdes et al.	2012	Prospective	28	RBES (peptic, anastomotic, corrosive, radiotherapy-induced, lichenplanus and other strictures)	90 days (range 14–618 days)	Not mentioned	Severe retrosternal pain with or without vomiting, hemorrhage, fever and aspiration pneumonia	7 (25%)	Hirdes et al. (2012a)
Griffiths et al.	2012	Prospective	24	Benign strictures (*n* = 7) and esophageal cancer (*n* = 17)	20 weeks (range 13–111) in benign group	0	Upper gastrointestinal bleed, nausea and vomiting, bronchopleural fistula	17 (77%)	Griffiths et al. (2012)
Hirdes et al.	2012	Prospective	19	MES (16 EAC and 3 ESCC)	51–140 days	1 (5%)	Severe retrosternal pain, nausea and vomiting, hematemesis and recurrent dysphagia	100%	Hirdes et al. (2012b)
Fischer et al.	2012	Case report	2	BES (restenosis after thoraco-abdominal esophageal resection for cancer and perforation)	12 months	0	Hypergranulation	1 (50%)	Fischer et al. (2012)
Dumoulin et al.	2012	Case report	1	RBES (high grade peptic stenosis in the distal esophagus)	4 months	0	Tissue hyperplasia	1 (100%)	Dumoulin and Plassmann (2012)
Basha et al.	2013	Case report	1	BES (corrosive-induced esophageal and pyloric strictures)	12 months	0	0	1 (100%)	Basha et al. (2013)
van den Berg et al.	2014	Prospective	10	MES (9 EAC and 1 ESCC)	93–166 days	0	Retrosternal pain, stent obstruction due to necrotic tissue development	10 (100%)	van den Berg et al. (2014)
McCain et al.	2016	Retrospective	20	9 RBES and 11 MES	At least 30 days	0	Post-procedural pain and failed deployment	Not mentioned	McCain et al. (2016)
Yano et al.	2017	Prospective	18	RBES (stricture after ESD, esophagectomy, or CRT of esophageal cancer)	24 weeks	0	Reactive hyperplastic nodules, esophageal pain, GERD, vomiting	12 (66.7%)	Yano et al. (2017)
Walter et al.	2018	Prospective	32	Recurrent BES (mainly anastomotic strictures)	12 months	1 (3.1%)	Stent occlusion, tracheoesophageal fistula, stent migration	Not mentioned	Walter et al. (2018)
Maishman et al.	2021	Prospective	12	MES (10 EAC and 2 ESCC)	52 weeks/until death	Not mentioned	Pain, vomit, esophageal or upper gastrointestinal hemorrhage	7 (58.3%)	Maishman et al. (2021)
Lopez-Tobaruela et al.	2021	Case report	1	BES (stricture in the upper esophagus)	2 weeks	0	Hepatic abscess	1 (100%)	Lopez-Tobaruela et al. (2021)
Yano et al.	2022	Prospective	30	BES (28 post treatment for esophageal cancer, 1 post-gastrectomy for gastric cancer, 1 congenital esophageal stricture)	6 months	0	Mucosal hyperplasia, esophageal pain, minor oropharyngeal pain, minor GERD, brain abscess	14 (46.7%)	Yano et al. (2022)
Kailla et al.	2023	Meta-analysis	246	BES (post-surgical, radiation, peptic, caustic and other strictures)	Not mentioned	6.5%	Recurrent dysphagia, severe thoracic pain, food bolus obstruction, tracheoesophageal fistula, esophageal ulceration, etc.	41.9%	Kailla et al. (2023)

ELLa-BDS has been extensively investigated in the management of MES, in addition to BES. In 2010, Stivaros et al. (2010) implanted two ELLa-BDSs in an octogenarian patient with cancer at the gastro-esophageal junction (GOJ). The study’s findings suggested that patients with dysphagia and potentially curable esophageal carcinoma could consider BDS as an alternative to gastrostomy, particularly in the context of neo-adjuvant or radical chemo/radiotherapy. Subsequently, a research group in 2012 treated 16 patients experiencing dysphagia from MES with ELLa-BDS (Griffiths et al., 2012). These patients included those scheduled to undergo neoadjuvant chemotherapy and planned esophagectomy (*n* = 9), those slated for radical radiotherapy with or without chemotherapy (*n* = 6), and those with metastatic esophageal cancer. This pioneering study represented the first exploration of BDS use in patients with a variety of malignancies. The results indicated that ELLa-BDS significantly alleviated dysphagia and reduced the need for enteral tubes in patients receiving radical chemoradiotherapy or awaiting esophagectomy. McCain et al. (2016) inserted ELLa-BDS in 11 patients with MES in 2016, further confirming the utility of ELLa-BDS in MES. Their study provided compelling evidence that BDS placement was a safe and effective adjunct in the management of MES. Importantly, it enabled the maintenance of enteral nutrition after staging or neo-adjuvant therapy, without adversely affecting subsequent surgical resection.

The insertion of an esophageal stent delivers instant relief in the palliative treatment of MES-induced dysphagia, whereas brachytherapy provides sustained relief (Hirdes et al, 2012b). A study conducted in 2012 assessed the impact of concurrent brachytherapy and ELLa-BDS insertion in 19 patients diagnosed with unresectable esophageal cancer (Hirdes et al, 2012b). This combination approach successfully restored lumen patency; however, it cannot be recommended for palliative esophageal carcinoma treatment owing to the increased likelihood of severe complications associated with the procedure. In 2014, van den Berg et al. (2014) investigated the safety and efficacy of ELLa-BDS as a surgical bridge preceding CRT in patients with dysphagia from locally advanced esophageal cancer. This study marked the first description of BDS insertion prior to neoadjuvant CRT in individuals diagnosed with locally advanced esophageal cancer. Importantly, no significant adverse incidents or mortality within 30 days associated with the procedure were observed, establishing the approach as safe and feasible. Individuals with esophageal cancer are at risk of developing RBES following ESD or CRT. Yano et al. (2017) confirmed that BDS is an efficient and well-tolerated therapeutic choice for RBES that occurs after esophageal cancer treatment, particularly following ESD or CRT; however, its long-term efficacy remains limited. In 2021, Maishman et al. (2021) employed the SIMON two-stage, single-arm, prospective phase II trial design to assess the efficacy of biodegradable stents in combination with radiotherapy in patients with esophageal cancer-related dysphagia who were not candidates for radical interventions. The observed elevated intervention rates indicate that the proposed alternative treatments may not be sufficiently efficacious to justify their inclusion in larger-scale trial designs. Therefore, additional research is required.

Furthermore, certain complications associated with ELLa-BDS treatment for BES have been documented. These include severe epithelial hyperplasia (Orive-Calzada et al., 2009; Hair and Devonshire, 2010; Dumoulin and Plassmann, 2012; Fischer et al., 2012), tracheoesophageal fistula (Jung et al., 2010), and hepatic abscess (Lopez-Tobaruela et al., 2021). In 2009, researchers inserted ELLa-BDS to address postoperative anastomotic stenosis in a patient (Orive-Calzada et al., 2009). However, the patient developed progressive dysphagia 3 months afterwards. Endoscopic examination showed that the stent had already degraded, and a new severe stenosis had developed, which was attributed to hyperplastic inflammatory tissue. This marked the inaugural report of this particular complication associated with ELLa-BDS, which was effectively managed using balloon dilation. In 2012, Dumoulin and Plassmann (2012) suggested argon plasma coagulation as another potential treatment option for this complication, considering its low risk of complications (Manner, 2008) and the alleviation of tissue hyperplasia once the stent is fully degraded.

In summary, ELLa-BDS demonstrates remarkable flexibility and biocompatibility. As previously mentioned, multiple studies have consistently confirmed the safety and efficacy of ELLa-BDS (van Boeckel et al., 2011; van Hooft et al., 2011; Hirdes et al, 2012a; McCain et al., 2016; Walter et al., 2018; Kailla et al., 2023). ELLa-BDS exhibited more promising results than PLLA-BDS, with a notably low migration rate (Dhar et al., 2009; van Boeckel et al., 2011; van Hooft et al., 2011; Yang et al., 2016). However, it has lower mechanical stability and a faster degradation rate than PLLA, typically undergoing complete degradation within 11–12 weeks. It should be emphasized that this rapid degradation can potentially lead to complications, such as the development of severe epithelial hyperplasia.

Han et al. (2018) designed a groundbreaking PDO and PLLA sheath-core biphasic monofilament to develop a novel BDS with enhanced mechanical characteristics and regulated biodegradability. This monofilament was further enhanced through functionalization with a conjugate of hyaluronic acid (HA) and dopamine (DA) and the addition of BaSO4 to enhance tissue adhesion and radiopacity. This pioneering stent design effectively harnessed PLLA’s relatively high mechanical properties, while mitigating the inherent drawback of PDO’s low mechanical stability during degradation. PDO/PLLA sheath-core monofilaments consistently exhibited superior long-term mechanical stability throughout the *in vitro* degradation assessment compared to PDO alone. Simultaneously, the stent combined DA’s outstanding binding properties with HA’s enhanced biocompatibility and anti-fouling effects. Furthermore, this design eliminated the need for stent removal and the associated trauma compared to non-degradable stents. Therefore, the performance of the newly developed biodegradable esophageal stent model in this study surpassed that of non-degradable stents and PDO stents. These findings hold significant relevance for future research in this field.

#### 2.1.4 PCL

PCL is an aliphatic polyester that belongs to the category of semi-crystalline polymers. It degrades relatively slowly and generates only minimal acidity during the degradation process (Rai et al., 2016). PCL is distinctive among other biodegradable polyesters owing to its commendable biocompatibility, elastic properties, resistance to fatigue, and cost-effectiveness (Little et al., 2009). PCL has been utilized in a variety of medical domains, including wound dressings, drug delivery devices, tissue engineering scaffolds, artificial blood vessels, and nerve regeneration devices (Sinha et al., 2004; Woodruff and Hutmacher, 2010; Abedalwafa et al., 2013; Mohamed and Yusoh, 2016). Furthermore, PCL is a highly promising candidate for use in drug delivery devices owing to its excellent biodegradability, mechanical characteristics, synthetic adaptability, compatibility with various drugs, and high drug permeability (Guarino et al., 2002; Labet and Thielemans, 2009; Rai et al., 2016; Washington et al., 2017; Thakur et al., 2021; Łukasiewicz et al., 2021).

Lei et al. (2010) employed 5-fluorouracil (5-FU) and PCL to produce multilayered films on esophageal stents. These films comprised a drug-free backing layer and a surface drug layer applied on the primary drug layer, allowing for a unidirectional and controlled drug release while enhancing mechanical characteristics. A series of studies focusing on the release and permeation behavior of the 5-FU-PCL multilayer proved the excellent performance of these multilayer films in the context of drug-controlled release stents. These flexible multilayer films exhibited the capacity to modulate drug release effectively and were regarded as a promising avenue for the development of DES. In 2013, Zhu et al. (2013a) (Zhu et al., 2013b) developed a novel biodegradable PCL-PTX nanofiber-covered metal stent. They evaluated its efficacy in managing benign cardiac stenosis in dogs, demonstrating that this innovative stent delivered ample radial force compared to its bare metal counterpart. Furthermore, PTX was steadily and continuously released through matrix diffusion and degradation, even with prolonged stent placement. This controlled release of PTX reduced inflammation and fibrosis and hindered scar tissue formation, ultimately improving disease prognosis. Notably, PTX exhibited a stable release duration under the acidic condition (pH = 4.0), lasting up to 32 days. *In vitro* studies showed that fibrous membranes containing a higher PTX concentration exhibited a more significant inhibitory impact on the proliferation rate of smooth muscle cells.

#### 2.1.5 PTMC

PTMC is a flexible amorphous polymer and a linear aliphatic polyester compound renowned for its remarkable elasticity and high toughness under room temperature and *in vivo* conditions (Jiang et al., 2022). Its exceptional biocompatibility and biodegradability (Hou et al., 2020), coupled with the absence of acidic degradation products during breakdown, effectively prevent severe inflammatory reactions (Yang et al., 2014; Yang et al., 2015). This versatility renders PTMC a valuable material in various biomedical applications, including drug delivery systems (Mohajeri et al., 2020; Hou et al., 2023) and tissue engineering (Li et al., 2020; Brossier et al., 2021; He et al., 2021), among others. In recent studies, researchers have explored hybrid polymers that combine PCL and PTMC as biodegradable coatings on Mg alloy stents, with promising results (Yuan et al., 2016). The growing attention on PTMC indicates its substantial potential for further advancements, despite its underutilization in esophageal stents.

### 2.2 Biodegradable Mg-based materials

Some studies have reported promising clinical trial outcomes for biodegradable polymer stents; however, they present with shortcomings, primarily concerning mechanical strength. Researchers have focused their attention on Mg alloys, a type of biodegradable metal, owing to their remarkable biocompatibility (Chen et al., 2018) and robust support capabilities. Mg alloys provide ample radial support force, particularly in cases of moderate to severe stenosis, effectively mitigating tissue hyperplasia (Gu et al., 2009; Li et al., 2015). Additionally, the degradation products of Mg alloys are alkaline, capable of neutralizing the acidic pH environment found at cancer sites, thus exhibiting the potential to inhibit cancer cells (Wang et al., 2019). Currently, Mg alloys have widespread applications in the biomedical field, including their use in fracture fixation, treatment of tracheal stenosis, and clinical deployment in coronary vascular stents, among others (Zhang et al., 2010; Perkins et al., 2015; Yue et al., 2015; Han et al., 2016). Nevertheless, a significant barrier that hinders the broad acceptance of Mg-based implants is their swift rate of degradation, with approximately 50% degradation occurring *in vitro* within just 1 week. This rapid degradation can result in the swift deterioration of the alloy’s mechanical properties, introducing difficulties in maintaining long-term structural support post implantation for effective disease treatment (Wong et al., 2010). Therefore, researchers in the field of biomaterials often focus on enhancing the corrosion resistance of Mg-based alloys, which is a critical advancement for the clinical application of Mg-based stents (Kirkland et al., 2010; Mueller et al., 2010). Some studies have explored methods involving the combination of Mg alloys with other metals or the coating of Mg alloys with non-degradable or degradable materials to address the limitations of pure Mg stents.

#### 2.2.1 Mg-Zn-Y-Nd alloy

Alloying strategies are frequently employed to enhance the properties of Mg alloys. Wang et al. (2020) investigated an Mg-Zn-Y-Nd alloy (Henan Key Laboratory of Advanced Magnesium Alloy, Zhengzhou, China), which has attracted substantial interest in the realm of cardiovascular stents (Chen et al., 2018; Wang et al., 2019). They investigated the viability of using this alloy as a biodegradable material for esophageal stents by conducting a comparative analysis of its mechanical properties against 317L stainless steel (a commercial non-degradable metal) and investigating the function of this alloy in inhibiting esophageal cancer cells. Their findings unveiled the promising potential of the Mg-Zn-Y-Nd alloy, primarily attributed to its low hardness and capacity to impede the growth of pathological cells associated with esophageal cancer. The integration of Mg alloy with advanced surface modification technologies may enhance the effectiveness of esophageal stents, thereby raising the standard of care for individuals with MES (Wang et al., 2020).

#### 2.2.2 Mg-polymer composites

The application of surface coatings on Mg alloys is essential for improving the performance of Mg-based stents. Currently, a variety of polymer materials have been widely employed to protect the surface of Mg alloys. These polymers include PLA (Chen et al., 2011; Li et al., 2018), PLGA (Liu and Xi, 2016; Chen et al, 2019b) and PCL (Chen et al., 2011; Zhang et al., 2016). Liu et al. (2022) focused on surface coating for esophageal stents and designed and tested a braided Mg-based stent coated with PLGA containing PTX, as previously described. This stent combined the strengths of Mg alloy and biodegradable synthetic polymer, with the additional benefit of PTX to inhibit tissue proliferation and prevent restenosis. This innovative approach has paved the way for further research, providing exciting opportunities to explore synergies between stents, coatings, and drug therapies to achieve functional improvements.

However, the acidic degradation products and general degradation behavior of the aforementioned polymers may potentially compromise the corrosion resistance of Mg alloy, rather than enhance it (Chen et al., 2011). Therefore, PTMC has garnered attention owing to its homogeneous surface-eroding behavior, which occurs uniformly from exterior to interior, and the production of charge-neutral degradation products. Its exceptional protective qualities render it a highly promising material for polymer coatings (Wang et al., 2013; Pan et al., 2020; Pan et al., 2022; Tang et al., 2022). Furthermore, silicon is a commonly utilized non-biodegradable coating that has garnered widespread applications in SEMS and SEPS.

**FIGURE 3:**
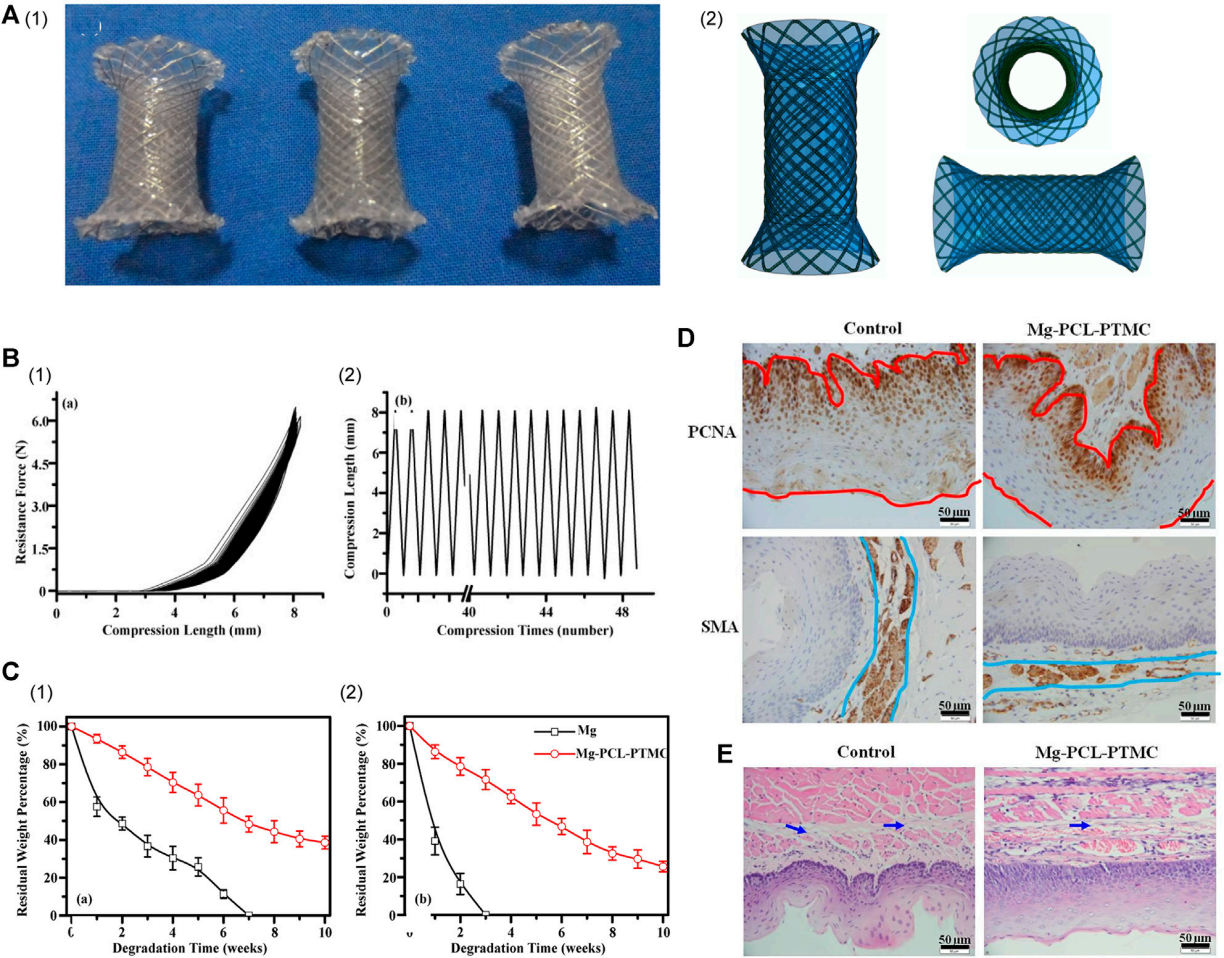
**(A)** (I) Photographs of the ideal expanded PCL-PTMC coated Mg-stent shape. (II) The covered membrane stent’s different profile diagrammatic cross-section. **(B)** (I) Mechanical compression curve analysis of the stent. (II) Compression-recovery curves of length and time in repeated compression tests (*n* = 5, constant pressure = 10 N). **(C)** Analysis of the degradation of the bare Mg and Mg-(PCL-PTMC) stent samples in phosphate-buffered saline with pH values of 7.4 (I) and 4.0 (II). **(D)** Both the epithelial and smooth muscle cell (SMC) layers were significantly thinner in the Mg-(PCL-PTMC) stent group than in the control group (Mg × 400, the red and blue lines indicate the thickness of the epithelial and SMA layers, respectively). **(E)** HE trichrome staining showed collagen deposition in the submucosa in the control and Mg-(PCL-PTMC) stent groups. *p* < 0.05 for comparisons between the control and Mg-(PCL-PTMC) stent groups. Reproduced with permission from ref [(Yuan et al., 2016)]. CC BY 4.0. Copyright 2016 The Author(s).

Several studies have applied coatings of the aforementioned polymers or silicon to enhance the performance of esophageal stents constructed from Mg-based alloys (Table 4). In 2016, Yuan et al. (2016) selected a blended polymer composed of PCL and PTMC for use as a biodegradable coating on Mg alloy (AZ31, Mg-3Al-1Zn) stents to develop a fully biodegradable esophageal stent. This innovative approach yielded stents characterized by exceptional flexibility, elasticity, and remarkable resistance to lesion compression, as validated through rigorous mechanical testing. The PCL-PTMC membrane played a pivotal role in significantly slowing the degradation rate of Mg alloys *in vitro*. *In vivo* research further emphasized the potential of these stents, illustrating their capacity to offer substantial support for a minimum of 4 weeks, devoid of any significant harm or excessive collagen accumulation. This breakthrough holds promise for clinical applications and offers a fresh perspective for the future development of biodegradable stents (Figure 3).

**TABLE 4:** Studies of Mg stents coated with membranes.

Author	Year	Animal model	Coating	Longest follow-up time	Effective support time	Degradation time *in vitro*	Technical success (%)	Migration (%)	Reference
Yuan et al.	2016	5 New Zealand rabbits	PCL-PTMC	4 weeks	4 weeks	10 weeks	5 (100%)	2 (40%)	Yuan et al. (2016)
Zhu et al.	2017	15 healthy rabbits	silicon	4 weeks	2 weeks	10 weeks	15 (100%)	6 (40%)	Zhu et al. (2017)
Yang et al.	2019	15 New Zealand rabbits with BES	silicon	3 weeks	2 weeks	10 weeks	15 (100%)	8 (53.3%)	Yang et al. (2019)
Liu et al.	2022	12 New Zealand rabbits with BES	PTX-PLGA	3 weeks	3 weeks	13 weeks	12 (100%)	7 (58.3%)	Liu et al. (2022)

Yang et al. (2019) (Zhu et al., 2017) developed an Mg stent coated with silicon, which possessed excellent flexibility and elasticity and exhibited impressive resilience to lesion compression during *in vivo* testing. In their study, the research team inserted the stents into the healthy esophagi of rabbits and observed that they offered sufficient radial force, with the silicon membrane significantly decreasing the rate of Mg biodegradation. Remarkably, the stent facilitated the remodeling of the esophageal wall with minimal tissue damage and elicited a minimal inflammatory response. Importantly, the stent proved capable of meeting crucial clinical requirements, including strength, safety, and reduced complications, with no esophagus injury or stent migration occurring in the rabbit experiments. Therefore, this silicon-coated Mg stent is expected to become a promising strategy for treating BES.

The fusion of Mg alloys with surface modification techniques bestows Mg-based materials with new surface attributes while preserving their intrinsic qualities. The application of suitable coatings onto the surface of Mg-based stents is a potent means to extend the degradation timeline of Mg-based implants, bolster their mechanical properties, and enhance their biological functionalities. Nonetheless, single-layer coatings might fall short of meeting the comprehensive performance requirements, including corrosion resistance and multiple functionalities. Therefore, future endeavors should focus on crafting composite coatings that harness the advantageous properties of each individual layer, thereby elevating the biological activity and adhesion of Mg-based stents (Zhang et al., 2021). In addition, the effective support duration of covered Mg stents remains limited, partly owing to a relatively high migration rate ranging from 40% to 58.3% (Yuan et al., 2016; Zhu et al., 2017; Yang et al., 2019; Liu et al., 2022). Addressing this issue necessitates further research focused on reducing the biodegradation rate and prolonging the duration of support. Extended follow-up studies are also essential for evaluating the efficacy of covered Mg stents, identifying the best time for insertion, and elucidating tissue reactions.

In summary, esophageal stents constructed from synthetic polyester polymers and Mg-based alloy materials have their own individual set of advantages and disadvantages, highlighting the need for further research to explore stents with improved performance. Currently, esophageal stents primarily serve the purpose of enlarging the esophageal lumen, particularly for addressing MES such as tumors. Coated SEMS have shown promise in reducing tumor cell ingrowth (Rozanes et al., 2002); however, they lack inherent anti-tumor or anti-proliferation capabilities. DES has been in development for many years; however, its research and application have been predominantly concentrated in the fields of coronary artery disease and peripheral artery disease, with research on esophageal stents being comparatively underexplored with slow progress. Stent migration is also a common problem with esophageal stents. Further technological innovation and animal and clinical trials are required to address these challenges. Additionally, the existing stent manufacturing technologies and materials are relatively limited, relying mainly on braiding technology, polyester polymers, and alloy materials. Urgent innovation is required to diversify and customize stent types, functions, and manufacturing processes.

## 3 Innovations and outlook

### 3.1 Functional innovation

#### 3.1.1 Unidirectional drug-release function

In recent years, DES has garnered increasing attention across various medical disciplines. It typically comprises three primary components: the stent body, the stent coating (generally composed of polymers), and an anti-proliferative drug (Garg et al., 2013). As previously mentioned, researchers are integrating antiproliferative drugs with stent coatings, applying them to either traditional SEMS or emerging BES. This approach preserves the primary function of the stent and facilitates the localized, sustained release of drugs. This effectively inhibits tissue proliferation in restenosis or tumor growth to attain the required local drug concentration, without necessitating potentially harmful systemic doses. Furthermore, some researchers have pioneered innovative drug-loading techniques to enhance the drug-release performance of DES. They have designed double-layer or multi-layer drug-loaded films that enable the unidirectional release of drug molecules onto the esophageal wall, ultimately achieving more precise and efficient treatment.

Guo et al (2007a) (Guo et al, 2007b) fully blended 5-FU particles and ethylene-vinyl acetate (EVA) in various proportions and compressed them into a film using a heat source to create a 5-FU-loaded layer. The drug-loaded layer was combined with a drug-free EVA protective layer, resulting in the development of an esophageal stent coating capable of unidirectional drug molecule release. Subsequently, they wrapped this coating around a nickel-titanium stent under controlled pressure at 70°C to form a 5-FU-coated stent. The findings indicated that regulating the permeation rate of 5-FU through the porcine esophageal mucosa was possible by adjusting the drug content within the coating. The coating containing 20%–60% 5-FU exhibited the ability to endure repeated binding and release through the stent introducer. In a subsequent study by the same team, they inserted the stent into the esophagi of healthy New Zealand albino rabbits. The results revealed that the 5-FU concentration in the esophageal tissue exposed to the stent, particularly the mucosal layer, consistently exceeded that in the serum or liver. This indicated that the stent had high efficiency and long-term local drug delivery capabilities and held the potential for the treatment of esophageal tumors and other tumors within the digestive and respiratory tracts (Guo et al., 2010). In 2015, Liu et al. (2015) (Wang et al., 2015) utilized the aforementioned approach (Guo et al, 2007b; Guo et al., 2010) to prepare nickel-titanium alloy stents. These stents incorporated a bilayered polymer film that contained either 5-FU or PTX. The researchers subsequently inserted these stents into the esophagi of healthy Bama mini-pigs for *in vivo* evaluation. The 5-FU stent and PTX stent did not induce significant damage to the esophageal wall at their insertion sites and did not exhibit obvious local or systemic toxicity. Furthermore, these stents efficiently suppressed ulceration, inflammation, and the growth of granulation tissue. These two DESs exhibited dual functionality as a stent and a localized drug delivery device, presenting a potential approach characterized by high effectiveness and an absence of systemic toxicity for treating esophageal cancer.

**FIGURE 4:**
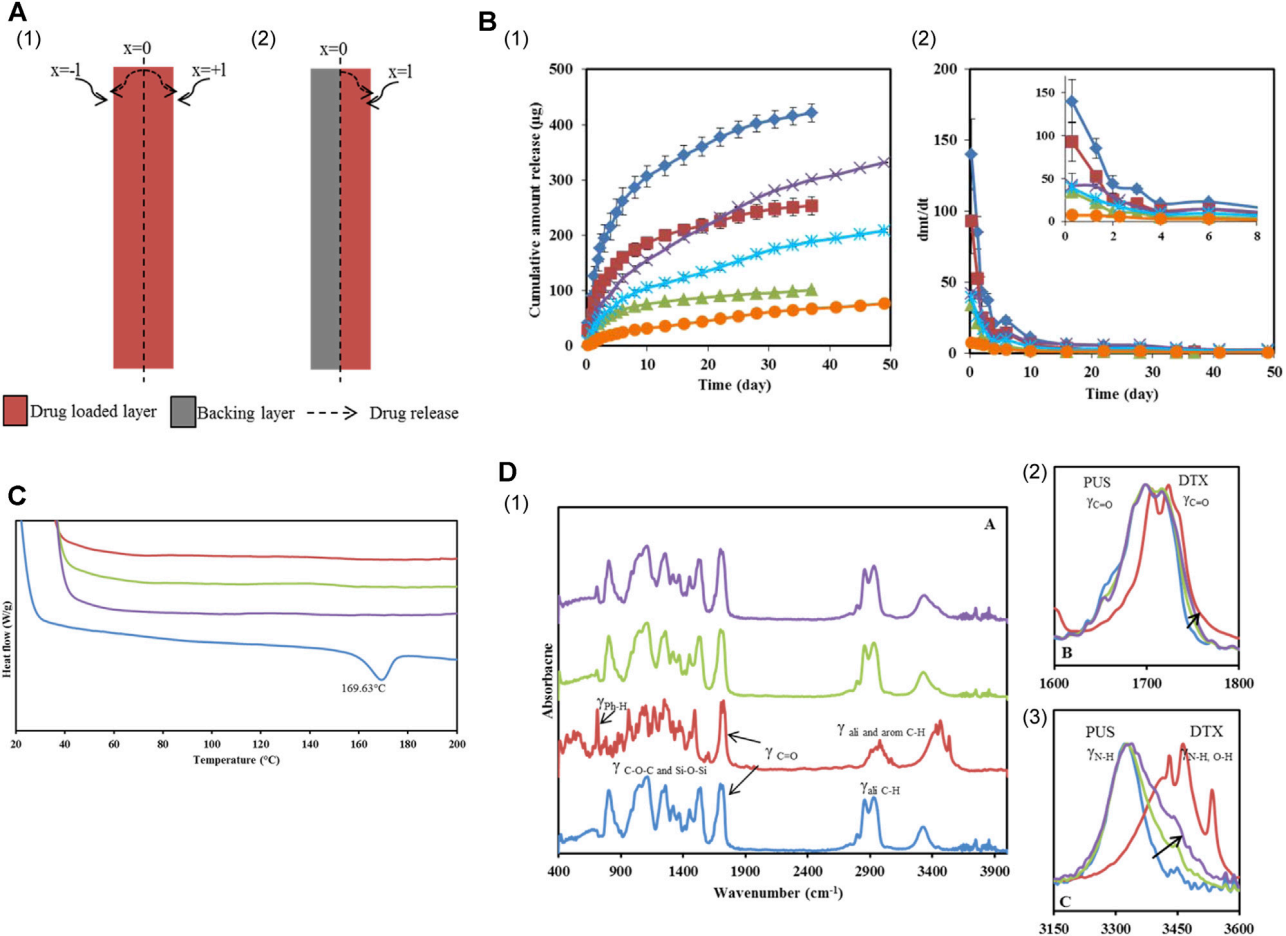
**(A)** Schematic illustration of DTX permeation through the mono and bilayer film. **(B)** *In vitro* DTX release profiles from Group 2 and 3 films. (I) Cumulative amount released and (II) Release rate from films loaded with different concentrations. **(C)** DES thermograms of PUS, 10% and 20% w/w DTX loaded PUS films and DTX. **(D)** (I) PA-FTIR spectra of PUS, DTX, 10% and 20% w/w DTX loaded films. (II and III) were the magnified spectra of the carbonyl and H-bond stretching regions. Reproduced with permission from ref [(Shaikh et al., 2015)]. Copyright 2015 American Chemical Society.

Researchers conducted experiments by incorporating varying concentrations of DTX into PurSil AL 20 (silicone-modified polyurethane polymer, PUS), which serves as a suitable material for covering SEMS, to achieve different loadings (Figure 4) (Shaikh et al., 2015). The solution was mixed evenly by vortex and ultrasonic, resulting in the formation of DTX-loaded films after the solution was dried. Researchers attached a blank film to the DTX-loaded film, creating a bilayer film, to ensure unidirectional drug delivery to the esophagus. Test outcomes demonstrated the physical and chemical compatibility of DTX and PUS, highlighting the outstanding physical and chemical stability of the DTX-loaded PUS membrane. The bilayer film ingeniously designed by the team exhibited sustained release properties (over 30 days) and held promise as a localized, continuous drug delivery system when combined with a stent.

In addition, Jin et al. (2018) introduced an innovative approach by designing and developing a novel antitumor drug/esophagus stent combination. This combination consisted of a magnetocaloric nitinol stent with a bi-layered film, which included one layer of ethylene-vinyl acetate copolymer (EVA) as a drug-blocking layer and another layer of EVA containing 10% PTX and 30% temperature-sensitive phase-change fatty alcohol (1-tetradecanol, 1-hexadecanol or 1-octadecanol). The stent exhibited excellent compatibility and safe magnetothermal drug release capabilities. The release rate of PTX from the PTX/nitinol complex accelerated upon subjection to an alternating electromagnetic field, resulting in a greater release amount. The penetration of PTX into the esophageal wall or deep esophageal musculature was significantly enhanced compared to that at 37°C. This experiment essentially combined a stent, an anticancer drug, and a phase-change material to enhance drug release and ease the penetration of drugs into esophageal tissue. This novel approach introduced a fresh stent design concept for managing esophageal cancer.

In conclusion, the introduction of drug-eluting esophageal stents and associated technological innovations has introduced new dimensions to the overall progress in esophageal stent development. Conducting valuable research aimed at improving drug-loaded polymers, refining drug coating manufacturing techniques, and performing associated investigations is crucial to further this advancement. Extensive studies of additional animals and patients are imperative to substantiate the efficacy and safety of these promising implants. Additionally, exploring alternatives such as DNA, siRNA, miRNA, and nanoparticles instead of traditional drugs for stent improvement, which is a field more extensively studied in cardiovascular stents (Zhao et al., 2018; Beshchasna et al., 2020), holds significant reference value for the ongoing development of esophageal stents.

#### 3.1.2 Anti-migration function

Due to the inherent physiological characteristics and peristaltic movements of the esophagus, coupled with the limited mechanical resilience of biodegradable stents, especially, there is a notable susceptibility to migration in biodegradable esophageal stents. Previous studies have indeed reported instances of early migration (Saito et al., 2007; Tanaka et al., 2007). Besides, despite the commendable mechanical properties of SEMS, the issue of stent migration persists, particularly in the case of fully-covered SEMS. Consequently, the innovation in the realm of anti-migration function emerges as a pivotal factor in enhancing stent performance.

In a noteworthy advancement in 2018, Lin et al. (2019) utilized 3D printing technology to fabricate esophageal stents with spirals. These stents exhibited substantial potential in mitigating the risk of migration, which will be elaborated upon later. Furthermore, in a recent patent, Clerc et al. (2022) introduced a novel approach by employing dissolvable or degradable adhesive polymers to fashion an anti-migration stent. This stent comprised both an inner and outer surface, with at least a portion of the outer surface incorporating a dissolvable or degradable adhesive polymer. The dissolvable adhesive polymer gradually dissolved in an aqueous environment over time, and the polymer layer was strategically positioned to come into contact with the inner surface of esophagus. Consequently, the activation of these polymer layers enhanced the adhesion of the self-expanding stent to esophagus, effectively resisting migration.

Moreover, it is crucial to design stents that not only minimize the extent of migration within the body lumen but also allow for easy removal and/or repositioning post-deployment. Hynes et al. (2022) introduced an expandable stent with distinct areas: a covered first section (facilitating removal) and an uncovered second section (reducing migration tendency). A biodegradable gripping material, featuring a roughened outer surface to resist post-implantation migration, was applied to at least one area of the biostable covering’s outer surface. The stent was designed to transition from a collapsed to an expanded state, with the second section intended to contact the inner surface of the body cavity in the expanded state.

While the stent designs outlined in the aforementioned patents have not been implemented in animals and clinical patients, their innovative approach to preventing migration has charted a new course for enhancing the performance of esophageal stent in future developments. It is anticipated that, leveraging these designs, multifunctional esophageal stents with enhanced performance can be developed.

### 3.2 Manufacturing technology innovation

Stent manufacturing technology has undergone continuous breakthroughs and advancements. Five main stent manufacturing methods are currently used: etching, micro-electro discharge machining, electroforming, die-casting, and the most commonly used laser cutting (Kathuria, 2006). Although laser cutting technology has matured, it still presents disadvantages such as high costs and the inability to cater to patients’ personalized customization needs (Wang et al, 2021a). Consequently, attention is shifting towards 3D printing technology, which is cost-effective, allows for personalized customization, and offers a broader range of material options.

3D printing is a digital manufacturing technology that utilizes bondable materials to construct objects layer-by-layer. Various 3D printing technologies, such as inkjet printing, stereolithography, selective laser sintering, and fused deposition modelling (Wang et al, 2021a), provide distinct advantages over traditional methods such as weaving, knitting, laser cutting, and segmentation. This innovative approach overcomes some of the limitations of current stent production and facilitates the customization and personalization of stent designs (Kang, 2019).

In 2018, a group utilized simulated structural parameters to 3D print four types of PLA/polyurethane (TPU) stents that incorporated spirals using a custom-made 3D printer equipped with four sets of PLA/TPU composites (Lin et al., 2019). Their findings indicated that 3D-printed stents with spirals displayed a significant potential for reducing migration risk and exhibited greater expansion force compared to stents lacking spirals. These stents degraded slowly, and their mechanical properties remained relatively stable even after 3 months of insertion, exhibiting the promising potential of 3D-printed esophageal stents in managing malignant esophageal diseases. Notably, this pioneering research marked the initial utilization of 3D printing technology for creating a polymer esophageal stent, showcasing the capability of this technique to produce esophageal stents with varying sizes and shapes. Further research is warranted to fully explore the functionality of 3D-printed esophageal stents (Kang, 2019).

In 2020, Fouladian et al. (2020) fabricated a 3D-printed DES using fused deposition modelling. This innovative stent featured a design where the central segment of the stent was constructed using PU filament loaded with 5-FU, whereas the two ends were constructed with drug-free PU filament. Test results indicated that this stent was capable of consistently releasing 5-FU over an impressive period of 110 days. Additionally, the stent exhibited remarkable stability, even when subjected to sterilization procedures involving gamma and ultraviolet irradiation and during accelerated storage conditions. This research demonstrated the effectiveness of 3D printing as a robust tool for constructing DES, which can be readily personalized to offer patient-specific geometries and precise drug dosages.

3D printing technology has seen limited application in the production of biodegradable esophageal stents; nevertheless, the results from previous experiments and its successful use in manufacturing other stent types are promising. We anticipate ongoing advancements and increased utilization of 3D printing technology to offer more tailored and precise treatments for esophageal stents, including DES with enhanced functionalities.

### 3.3 Material innovation

Hydrogels arising from the physical or chemical cross-linking of polymer chains represent hydrophilic polymers characterized by a three-dimensional porous structure (Zhang and Khademhosseini, 2017; Liu et al., 2018). They possess a soft and pliable texture, excel in retaining water, and exhibit commendable biocompatibility (Cai et al., 2022). Hydrogels have promising applications across various biomedical fields (Ustürk et al., 2022), including drug delivery systems (Zhang et al., 2019; Kasiński et al., 2020), cell culture substrates (Fukunaga et al., 2019), wound dressings (Mandal et al., 2020), and tissue engineering scaffolds (Neves et al., 2020; Unal and West, 2020).

**FIGURE 5:**
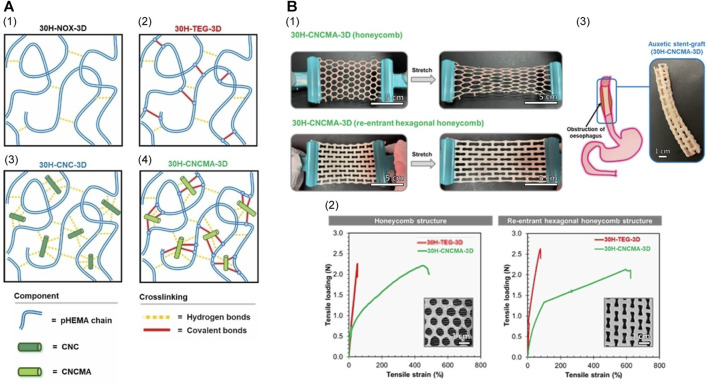
**(A)** Proposed schematic cross-linking networks for each hydrogel system. **(B)** Mechanical properties of pHEMA hydrogel specimens with conventional honeycomb and auxetic re-entrant hexagonal honeycomb structures. (I) Demonstration of the conventional and auxetic CNCMA-HEMA hydrogel specimens. The auxetic hydrogel specimen was expandable in the lateral direction when stretched while the conventional hydrogel specimen contracted. (II) Tensile load-strain curves of pHEMA hydrogel specimens with conventional and auxetic structures. (III) Esophageal stent made from auxetic CNCMA-HEMA hydrogel. Reproduced with permission from ref [(Pruksawan et al., 2022)]. Copyright 2022 Wiley-VCH GmbH.

In 2020, Raman et al. (2020) constructed a tough acrylate ortho-nitrobenzyl (oNB) light-triggered degradable hydrogel with adjustable mechanical characteristics and a modular design that can undergo safe, contact-free degradation at various anatomical sites within the body. The material and its degradation by-products were shown to be biocompatible. This group constructed an oNB esophageal stent using this light-triggerable hydrogel in combination with PCL, envisioning its application for offering structural support and/or localized drug delivery within the esophagus. Results from a series of *in vitro* and *ex vivo* trials suggested that these stents would persist in the esophagus when deployed *in vivo* until they naturally biodegrade. Subsequently, peristaltic waves within the gastrointestinal tract would gently transport the degraded material, eliminating the need for mechanical, thermal, or chemical triggers and minimizing adverse effects on the esophagus. This light-triggering mechanism provided a non-contact approach for the safe extraction of the stent from the lower esophageal sphincter, rendering it suitable for addressing both benign and malignant strictures. In a recent study, researchers developed CNCMA-pHEMA hydrogels using methacrylate cellulose nanocrystals (CNCMAs) as a macro-cross-linking agent in poly (2-hydroxyethyl methacrylate) (pHEMA) hydrogels (Figure 5) (Pruksawan et al., 2022). These hydrogels exhibited significantly enhanced toughness and tensile properties compared to hydrogels cross-linked with conventional agents. The remarkable strength and durability of these hydrogels render them promising candidates for advanced flexible implantable devices, including biodegradable esophageal self-expanding stents. Consequently, biodegradable hydrogels hold tremendous potential for the development of such stents.

### 3.4 Outlook

Biodegradable esophageal stents have garnered significant attention owing to their favorable biocompatibility and unique advantages, including the avoidance of secondary interventions. These stents have been employed in numerous animal experiments and clinical applications. However, biodegradable stents lag behind metal stents in terms of stiffness and strength. This discrepancy can lead to complications such as early displacement. Additionally, they are susceptible to the acidic environment in the esophagus, hastening the degradation process. The development of a more optimal biodegradable stent remains a challenge, despite the abovementioned ongoing innovative research in functions, technologies, and materials. Future research should delve more deeply into the material’s mechanical properties, degradation time, and surface characteristics to address these issues.

## 4 Conclusion

Biodegradable esophageal stents approved for clinical use, such as PLLA-BDS, ELLa-BDS, and other stents manufactured from synthetic polyester polymers and Mg-based alloys, have shown promising results in performance tests and clinical applications. However, this is only the beginning of the era of biodegradable esophageal stents, and the stents currently available in the market require further improvement regarding mechanical stability and material degradation rate, among others. Another promising characteristic of stents is their exceptional capacity to transport and locally release drugs to inhibit tissue proliferation or exert antitumor effects. These drugs can be eluted as the stent itself or the coating degrades, assisting in the treatment of stenosis or eliminating the cause of stenosis, with minimal systemic side effects. In addition, unidirectional drug-release function, anti-migration function, 3D printing technology and hydrogel materials are currently valuable innovations that deserve further exploration to develop better biodegradable esophageal stents.

In summary, the development of biodegradable esophageal stents remains in its initial exploration stage. It is anticipated that this field will witness the application of more appropriate biodegradable materials, enhanced and personalized stent manufacturing methods, improved stent structure and performance, and more precise drug-release technology.

## Abbreviations

ES, esophageal stricture; BES, benign esophageal stricture; MES, malignant esophageal stricture; RBES, refractory benign esophageal stricture; GERD, gastroesophageal reflux disease; ESD, endoscopic submucosal dissection; EMR, endoscopic mucosal resection; ESCC, esophageal squamous cell carcinoma; EAC, esophageal adenocarcinoma; SEMS, self-expanding metal stents; SEPS, self-expanding plastic stents; BDS, biodegradable stents; FDA, the US Food and Drug Administration; PLA, polylactic acid; PLLA, poly (L-lactic acid); PLGA, poly (lactic-co-glycolic acid); PDO, polydioxanone; PCL, poly (ε-caprolactone); PTMC, poly (1, 3-trimethylene carbonate); 5-FU, 5-fluorouracil.
